# Biting rhythm and demographic attributes of *Aedes albopictus* (Skuse) females from different urbanized settings in Penang Island, Malaysia under uncontrolled laboratory conditions

**DOI:** 10.1371/journal.pone.0241688

**Published:** 2020-11-11

**Authors:** Nor Atikah Farhah Muhammad, Nur Faeza Abu Kassim, Abdul Hafiz Ab Majid, Azimah Abd Rahman, Hamady Dieng, Silas Wintuma Avicor

**Affiliations:** 1 School of Biological Sciences, Universiti Sains Malaysia, Minden, Penang, Malaysia; 2 Vector Control Research Unit, School of Biological Sciences, Universiti Sains Malaysia, Minden, Penang, Malaysia; 3 Household and Structural Urban Entomology Laboratory, Vector Control Research Unit (VCRU), School of Biological Sciences, Universiti Sains Malaysia, Minden, Penang, Malaysia; 4 School of Humanities, Universiti Sains Malaysia, Minden, Penang, Malaysia; 5 Institute of Biodiversity and Environmental Conservation (IBEC), Universiti Malaysia Sarawak, Kota Samarahan, Sarawak, Malaysia; 6 Entomology Division, Cocoa Research Institute of Ghana (CRIG), New Tafo-Akim, Eastern Region, Ghana; National Taiwan Ocean University, TAIWAN

## Abstract

Urbanization could potentially modify *Aedes albopictus’* ecology by changing the dynamics of the species, and affecting their breeding sites due to environmental changes, and thus contribute to dengue outbreaks. Thus, this study was conducted to evaluate the biting rhythm, fecundity and longevity of adult female *Ae*. *albopictus* in relation to urbanization strata; urban, suburban and rural areas in Penang Island, Malaysia. The experiments were done in comparison to a laboratory strain. Twenty-four hours biting activity of all the mosquito strains showed a clear bimodal biting activity, with morning and evening twilight peaks. The interaction effect between biting time and mosquito strains was not significant. Meanwhile, differences in fecundity among mosquito strains were statistically significant (F(3,442) = 10.559, P < 0.05) with urban areas having higher mean number of eggs (mean = 107.69, standard error = 3.98) than suburban (mean = 94.48, standard error = 5.18), and rural areas (mean = 72.52, standard error = 3.87). Longevity of adult females were significantly higher (F(3,441) = 31.259, P < 0.05) for mosquito strains from urban areas compared to the other strains. These findings would provide crucial information for the planning of control programs in Malaysia, particularly Penang.

## Author summary

*Aedes* mosquito populations associated with human habitation in urban areas do not only have the potential to cause biting nuisance, but also cause significant public health risks through the transmission of dengue virus. The socioeconomic effects of urbanization have been comprehensively studied by socio-ecologists, but the ecological effects and their impact on this vector’s biology is not known. The authors found that in Penang Island, the mean number of eggs laid per female of *Aedes albopictus* was high in the urban area than those in the suburban and rural areas. Survivorship was high for urban populations parallel to fecundity and the apparent biting pattern which peaked at dawn and dusk was noted for all *Ae*. *albopictus* strains. The changed environment in the urbanized area where more types of breeding containers and more blood sources produced by condensed human population supported by warm climate may facilitate larval development and enhance vector survivorship and reproductive fitness. These might be the reasons for quick adaptation of *Ae*. *albopictus* in urban areas. As higher fecundity and longer adult survival may enhance disease transmission, attention on this species is needed in terms of vector control.

## Introduction

Urbanization primarily results in the physical growth of urban areas, leading to environmental changes. It is a global trend that results from economic development. Malaysia is among the fastest growing developing countries in the East [1], and the unprecedented movement of people into these areas is predicted to accelerate in the future [2]. A lot of problems have arisen as a result of urbanization and Malaysia is no exception. Among the problems that often take center stage in urbanization debates include environmental pollution, space, population density and the destruction of natural ecology [3]. The socioeconomic effects of urbanization have been comprehensively studied by socio-ecologists [4–6]. However, the ecological effects and their impact on vector biology and vector-borne infectious disease transmission remain unclear.

Most of the previous studies were focused mainly on oviposition ecology from larval habitats and abundance of *Aedes* species in various urbanized settings [3,7,8]. However, the biting activity, fecundity and survivorship of individual female *Ae*. *albopictus* are unknown in response to urbanization level particularly in Penang Island. In order to determine how environmental changes due to urbanization affect the life history traits of *Ae*. *albopictus* in terms of survival and reproductive fitness, this present study was conducted to determine the biting rhythm, egg production and longevity of adult female *Ae*. *albopictus* strains from Jelutong (urban), Batu Maung (suburban), and Balik Pulau (rural) in Penang Island in comparison to a laboratory strain. Since immature mosquitoes are sensitive to environmental changes [3,9,10], we hypothesized that urbanization increases *Ae*. *albopictus* eggs production and adult survivorship. The findings would provide valuable information on *Ae*. *albopictus* and help in improving current vector control and surveillance strategies for dengue that are adapted for specific settings in Penang Island.

## Materials and methods

### Study sites

The ovitrap sampling was conducted in three different areas which represent urban, suburban and rural settings in Penang Island. The distance between each area is approximately 21 km.

The urban location chosen for this study was Jelutong (5°23'15.9"N 100°18'29.2"E), which is located at the Northeast Penang Island District. The attributes of this area which include high human population density, little vegetation with the land use types being primarily residential and commercial buildings which are close to each other qualify an urban area [11].

The selected suburban location was Batu Maung (5°17'14.5"N 100°16'59.5"E), an area with land use that includes a mixture of residential, manufacturing, and several small fishing villages as well as a small number of scattered vegetation, less buildings and population density but greater house yards in comparison to an urban area [11].

The rural area was Kampung Pulau Betung in Balik Pulau (5°18'13.4"N 100°11'54.0"E), which is located near a fishing ground with an economy based on fishing. It is a large and open area which is isolated from the city with high vegetation cover and fewer houses compared to urban and suburban areas due to a low human population density [11].

Both Batu Maung and Balik Pulau are located at the Southwest Penang Island District. All sample collections were done on public land and the laboratory strain of *Ae*. *albopictus* was retrieved from the Vector Control Research Unit (VCRU), Universiti Sains Malaysia (USM).

### Mosquito sampling and rearing methods

In this study, *Ae*. *albopictus* were collected as larvae (fourth instar) and pupae from the three study sites. A total of 30 ovitraps were deployed randomly at two meters apart throughout the respective study areas at shaded sites to maximize the attractiveness of females to oviposit [12]. Each of the ovitraps was filled with approximately 250 ml of chlorine-free water and a paddle was placed as an oviposition substrate for the mosquitoes to lay eggs. Five days later, samples from the ovitraps were placed into plastic bottles and brought back to the insectary for rearing. Only larvae (fourth instar) and pupae samples were transferred into paper cups as temporary containers containing water from the ovitraps used and the top of the cup surface was covered with nylon netting. The pupae were reared until adult emergence. The eggs from the laboratory strain which were retrieved from the VCRU were reared until adult emergence to be used in the experiments as well. Mosquitoes were reared under 28.6 ± 1.8°C and relative humidity of 65–80%.

Throughout this study, temperature and relative humidity in the insectary were allowed to fluctuate with the weather outside which is an uncontrolled condition. Photoperiod was also unregulated and changed with the surrounding environment. Windows were opened and neither temperature controller nor air conditioner was used during this study.

### Mosquito colonies

Upon emergence, adults were identified based on basic identification keys (dorsal features) following Rattanarithikul and Panthusiri [13]. Only *Aedes albopictus* species were transferred into a standard mosquito rearing cage (30 cm x 30 cm x 30 cm) covered with nylon netting accordingly. For each mosquito strain (urban, suburban, rural and laboratory), 40 females and 40 males aged 3 to 5 days old were placed simultaneously in a cage. The mosquitoes were given access to a small cotton wool soaked in 10% sucrose solution on the first day of their emergence and were replaced every two to three days in order to avoid any fungal growth. The mosquitoes were allowed to freely mate for two days, and thereafter starved for a brief period (12 h) [14] before the experiments were executed. Animal Research and Service Centre (ARASC); USM/Animal Ethics Approval/2016/(101)(820) has specifically approved this study.

### Experimental design

#### (i) Biting rhythm

The first experiment was conducted to determine the biting rhythm of *Aedes albopictus* females from the four different strains within 24 hours. On the day of the experiment, the female mosquitoes were offered a restrained mouse in a narrow and fine wire-mesh cage, at 20:30 h, which is 1 h after sunset when *Ae*. *albopictus* is reported to be almost inactive [15,16]. The experiment was ended at 20:30 h on the next day. In approval of animal ethics from The Animal Ethics Committee, USM, the mosquitoes were offered with continuous blood meal for 24 hours, and the mouse was changed every four hours. Engorgement was checked at 24 time points (21:30, 22:30, 23:30, 24:30, 01:30, 02:30, 03:30, 04:30, 05:30, 06:30, 07.30, 08:30, 09:30, 10:30, 11:30, 12:30, 13:30, 14:30, 15:30, 16:30, 17:30, 18:30, 19:30 and 20:30) and the feeding time was recorded for each female. The fully engorged females resting on the walls of the cage were removed and transferred into another cage in order to avoid confusion. The experiment was repeated three times as replicates for each mosquito strain (80 mosquitoes per replicate: 40 females and 40 males).

#### (ii) Fecundity and longevity

The second and third experiments were conducted to determine the egg production and longevity of *Aedes albopictus* adult females from the four different strains. The female mosquitoes as mentioned in the **sub-section Mosquito colonies** were offered a restrained mouse in a narrow and fine wire-mesh cage for one hour; 18:00–19:00 hours, the closest time to the peak biting activity of *Ae*. *albopictus* in the field [17].

A day after, females were considered gravid [12]. Every gravid female was then placed singly into an oviposition cage (1.3 L) consisting of a modified plastic bottle supplied with a small cotton wool soaked in 10% sucrose solution through a thin nylon cloth covering the top end of the bottle. Cotton wools were replaced every two to three days. A small disposable plastic cup filled with 30 ml of chlorine-free water and lined with filter paper as an oviposition substrate was placed in each cage to provide females with sites for egg deposition. The filter paper was folded into a double-chambered cone and placed so that the mosquitoes could lay their eggs inside or outside the cone [14]. The separation of the females into individual oviposition cages facilitated the recording of the number of eggs produced by each individual female.

Two days later, egg deposition was checked daily and filter paper with eggs was removed. Eggs were counted under a dissecting microscope and a new filter paper was placed in each cage. This process was repeated until the death of the female. In order to determine the longevity of the *Ae*. *albopictus* females, the dead mosquitoes were recorded and removed from the cage daily. Thus, the computed longevity value represents the longevity of only the adult stage. The experiment was repeated three times as replicates for each mosquito strain (80 mosquitoes per replicate: 40 females and 40 males).

Throughout the series of experiments involved in this study, temperature and relative humidity in the insectary were allowed to fluctuate with the weather outside which were uncontrolled conditions in order to imitate the actual surrounding environment of mosquitoes [12]. Photoperiod was also unregulated and changed with the surrounding environment. Windows were opened and neither temperature controller nor air conditioner was used during this study.

### Data analysis

SPSS version 20.0 was used for statistical analyses. Data were tested for normality using Shapiro-Wilk statistics (P > 0.05). Data were transformed appropriately in case of non-normal distribution.

For biting rhythm, the number of fully-engorged mosquitoes (dependent variable) from the four different strains in relation to blood feeding time (independent variables) was subjected to two-way analysis of variance (ANOVA) to examine the effect of time and mosquito strain on biting rhythms of *Ae*. *albopictus* females.

Whereas, for fecundity, the number of eggs produced per mosquito (dependent variable) was used to compare between mosquito strains (independent variable) and was analyzed using one-way ANOVA followed by Tukey’s multiple comparison test.

Meanwhile, Kaplan-Meier survival analysis along with a log-rank test was performed to estimate the longevity of *Ae*. *albopictus* adult females from each strain. A log-rank test was run to determine if there were differences in the survival distribution for the four different mosquito strains.

## Results

### Biting rhythm of *Aedes albopictus* females from four different strains; urban, suburban, rural, and laboratory

**Fig 1:**
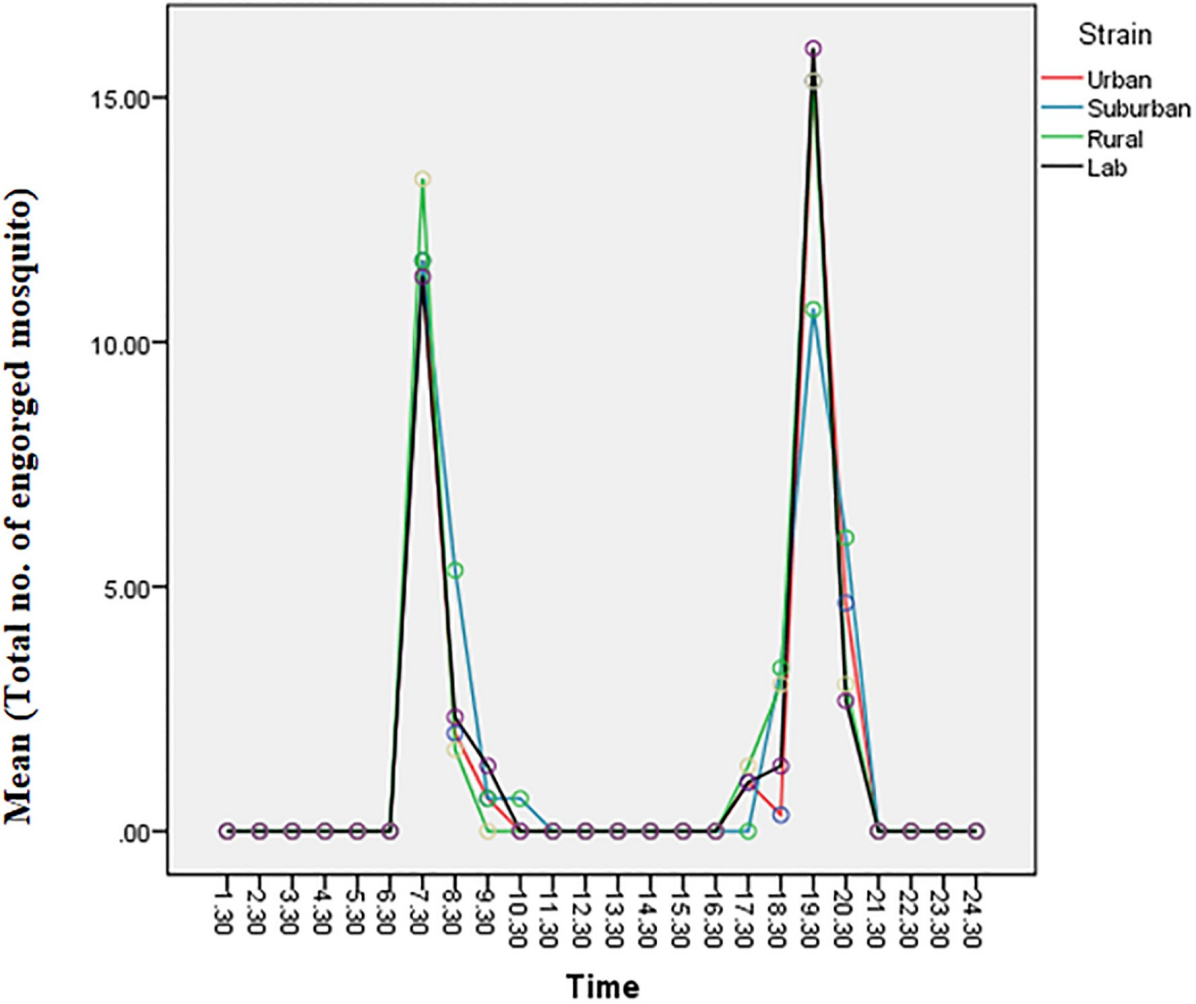
Biting rhythm of *Aedes albopictus* females from four strains; urban, suburban, rural, and laboratory.

Fig 1 shows the result of the 24 h biting cycle experiment performed under uncontrolled laboratory conditions. The experiment revealed two activity peaks for *Aedes albopictus*, coinciding with crepuscular dawn, 06:30–09:30 hours, and dusk, 18:30–20:30 hours for all four mosquito strains.

There was statistically no significant interaction between the effects of biting time and mosquito strains (F(69,192) = 1.337, P > 0.05) on the number of engorged *Ae*. *albopictus* female mosquitoes. Thus, *Ae*. *albopictus* biting cycle remained as a bimodal pattern regardless of the strain of mosquito [15,18]. Aside bimodal, the biting behavior is crepuscular, thus the insects are active primarily during twilight [19].

### Fecundity of *Aedes albopictus* females from four different strains; urban, suburban, rural, and laboratory

**Fig 2:**
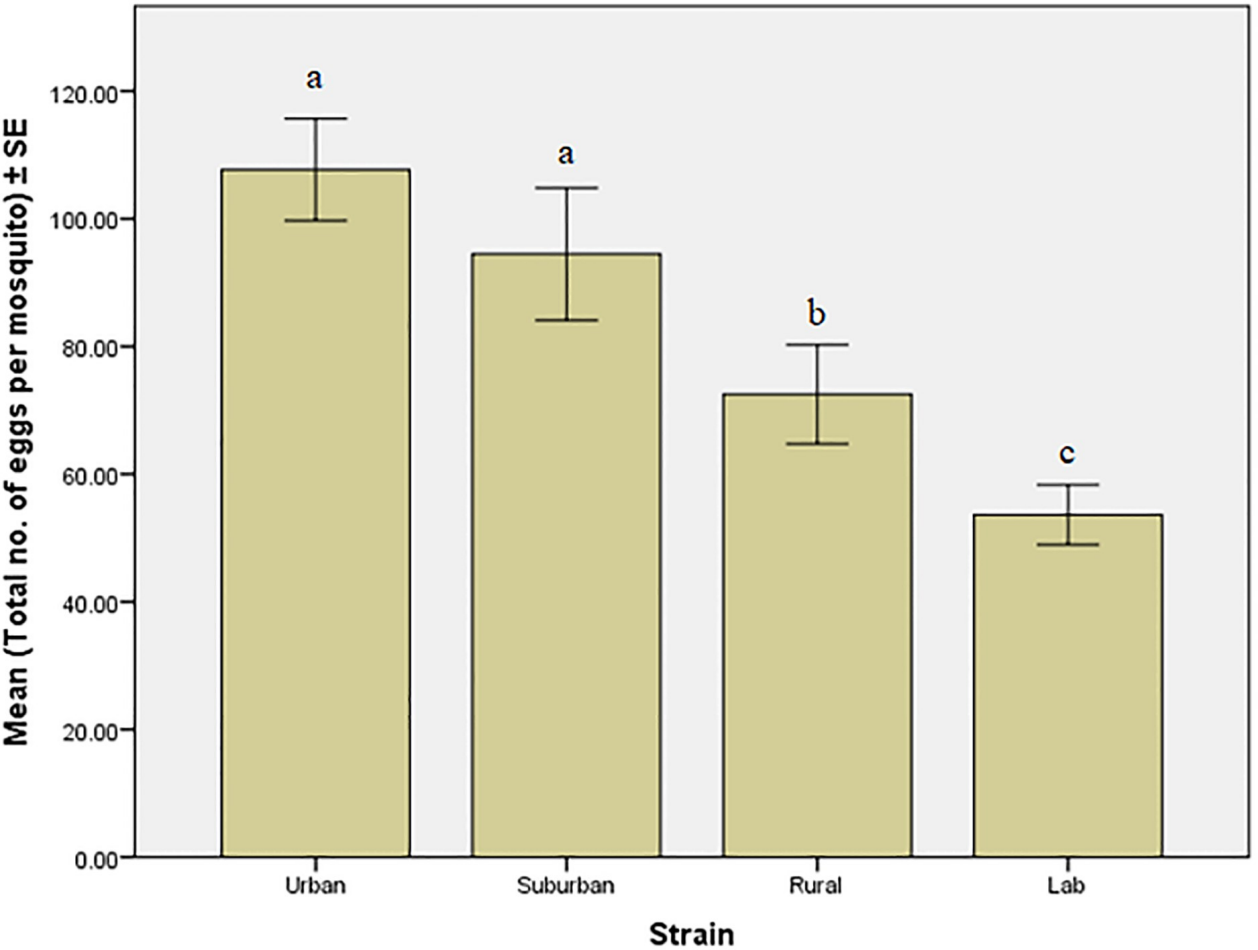
Total number of eggs produced by individual *Aedes albopictus* females in four different strains. **the same letter indicated no significant difference between strains (P > 0.05). **different letters indicated significant difference between strains (P < 0.05).

Based on the fecundity test conducted, the mean number of eggs produced by *Ae*. *albopictus* increased from the laboratory to urban areas. Referring to Fig 2, females from the urban area showed the highest mean with 107.69 ± 3.98 number of eggs produced per mosquito compared to those in the suburban (94.48 ± 5.18), rural (72.52 ± 3.87) and laboratory (53.65 ± 2.34) strains.

There was a significant difference in egg production between strains (F(3,442) = 10.5, P = < 0.05). Tukey’s multiple comparison test further showed that fecundity for urban and suburban mosquito strains was significantly different from rural and laboratory strains (P < 0.05).

### Longevity of *Aedes albopictus* females from four different strains; urban, suburban, rural and laboratory

**Fig 3:**
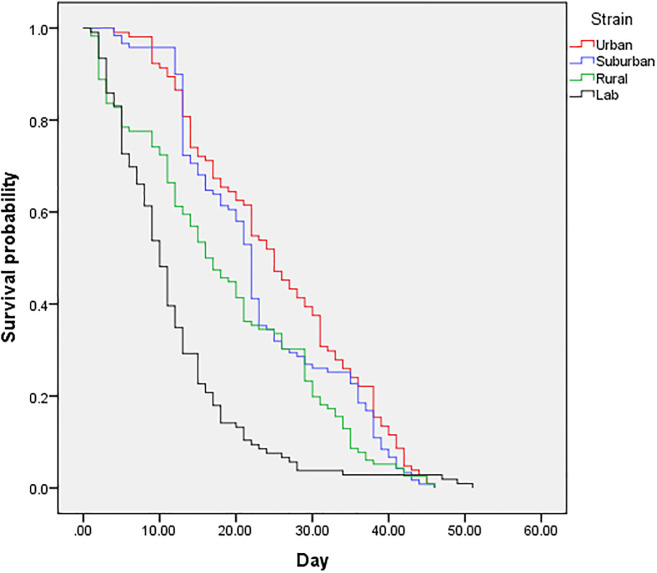
Cumulative survival probability of *Aedes albopictus* females from four different strains.

The survival analysis shows distinct differences in the longevity of *Ae*. *albopictus* adult females between the four strains (urban, suburban, rural and laboratory). Referring to Fig 3, females from the urban area showed the highest mean days of lifespan with 25.29 ± 1.10 than those in suburban (23.12 ± 0.97), rural (18.60 ± 1.14), and laboratory (11.89 ± 0.90) strains.

A log-rank test showed significant difference in the survival distributions for the four different mosquito strains (*X*^*2*^ = 72.28, d.f. = 3, P < 0.05).

## Discussion

Based on the 24 h biting activity of all the mosquito strains, *Ae*. *albopictus* females showed a clear bimodal activity, with morning (06:30 h to 09:30 h) and evening twilight (18:30 h to 20:30 h) peaks, similar as observed elsewhere in Southeast Asia and South Asia through human landing catch technique in the field [15,18,20–22]. However, we observed the biting activity under uncontrolled laboratory conditions, where *Ae*. *albopictus* females were offered a restrained mouse as their blood meal at 20:30 h. When we checked for engorgement at the next hour in accordance with the time points, surprisingly, blood-feeding and host-seeking activities were inactive. The same situation happened for almost ten hours of the experiment (21:30 h to 06:30 h). No engorged mosquitoes resting on the cage wall were recorded within that duration. Our finding was supported by Chen *et al*. [21] which showed biting peaks for *Ae*. *albopictus* at 07:00 h to 09:00 h and 17:00 h to 19:00 h. Chen *et al*. [22] also reported on the biting activity in urban residential areas in Kuala Lumpur was detected throughout the day, but the biting peaked between 06:00 h to 09:00 h and 15:00 h to 20:00 h, and had low biting activity from late night until the next morning (20:00 h to 05:00 h). In general, biting activity of Aedes was active throughout the day and night but no biting activities were recorded after 23:00 at any study site [22].

This behavior might simply explain the biting nature of this species. According to Kawada *et al*. [23], the nocturnal host-seeking activity positively correlated with the increasing light intensity as *Ae*. *albopictus* is sensitive to dim light. In their study, they found out that the host-seeking activity for this species is deactivated in complete darkness even during the daytime, irrespective of their increasing flight activity controlled by their intrinsic circadian rhythms [23]. The host-seeking activity changes with variation in the light intensity during the scotophase (0 to 100 lux) [23]. The rapid drop in activity level following the onset of darkness suggests that light promotes activity or, less likely, that the absence of light reduces the activity. Light may have a direct effect in determining the amount of activity and indirect effect through setting the phase of the endogenous rhythm [24]. The nocturnal feeding might not be due to the evolutionary adaptation to light, but by the intrinsic reaction of mosquito to light [23]. Thus, the potential risks of transmission possibilities of dengue virus exist wherever the vector inhabits (urban, suburban or rural areas).

Previous studies were mainly on oviposition ecology and abundance of species as influenced by urbanization [3,7,23] without knowing how many eggs one female could produce in a lifetime. By increasing eggs production by *Aedes* mosquitoes, urbanization could potentially worsen the epidemic risk factors for arboviruses. An increase in *Ae*. *albopictus* prevalence and abundance by urbanization was reported by [3] and it was stated that this phenomenon is probably due to elevated numbers of *Aedes* breeding sites such as tires, discarded cans or water storage containers, provided by urbanizing environments [3]. They reported that an urbanized environment accelerates *Aedes* mosquito development and survivorship. A deeper understanding of the modifications induced by urbanization in the ecology of *Ae*. *albopictus* is indispensable to improvise vector control strategies in the future.

In the present study, fecundity or mean number of eggs laid per female of *Ae*. *albopictus* in the first gonotrophic cycle was high in the urban area (107.69 ± 3.98) than those in suburban (94.48 ± 5.18), and rural areas (72.52 ± 3.87). This finding is supported by a study done by [25] where they found that *Aedes aegypti* which exist in forests had lower reproductive potentials than the typical form which lives in urban and suburban areas. Therefore, they speculated that the forest probably provides a more homogenous environmental stress than the urban habitat, where urban populations have adapted to a fluctuating environment which is probably related to variation in the availability of larval habitats, food source, temperature and humidity. Egg production in the field is dependent on environmental factors such as temperature and relative humidity since these factors influence the survival of adult mosquitoes. Warmer temperature in urban areas will lead to increased human biting frequency and subsequently accelerate the digestion of blood meals taken by mosquitoes, and eventually increased fecundity and reproductive fitness [26]. Apart from that, the number of eggs laid by *Ae*. *albopictus* females also depends on the physiological age, the body weight after emergence and particularly the size of the blood meal [27]. The development of the immature stages may influence egg production too. An optimum condition during the growth of immatures will result in larger and healthier adults who can consume more blood from the hosts [12]. The amount of blood imbibed and the blood digestion rate by the female determines the number of eggs produced. More eggs will be produced when an adequate amount of blood is consumed and rapidly digested [28].

These results reflected in the abundance of pupal and larval density in urban rather than suburban and rural areas [3,29]. This might be due to the fact that urban areas had less predators, more nutrition from a “dirtier” environment, or even less drift from agricultural insecticides [3]. The pupal productivity is a good indicator of the abundance of adult mosquitoes [30–32].

Higher mosquito fecundity does not necessarily lead to increased disease transmission if adult mosquitoes have a very short life span. This is because longevity must be sufficiently long in order to allow for the development of pathogens in their bodies to be completed. We found that female mosquitoes in urban areas had the longest life span compared to the other two areas. This result may be due to environmental factors such as temperature and humidity, where the average temperature in urban areas is higher than in suburban and rural areas [3]. This finding is similar to a previous study by [33], where they found that the median survival of *Anopheles arabiensis* in the deforested area was 49 to 55% higher than those in the forested area and the net reproductive rate of female mosquitoes in the deforested area was 1.7 to 2.6 fold higher than that in the forested area in the East of Africa. Therefore, they concluded that deforestation or urbanization enhanced the survivorship of adult mosquitoes [33].

Longer adult female longevity may enhance disease transmission, although the exact correlation between vector capacity and adult life span needs to be further explored. In this study, we fed the female mosquitoes with 10% sucrose solution without blood, which might have exerted stress on the females during the gonotrophic cycle and affected the longevity of the female mosquitoes [12]. Thus, we predict that the females will live much longer if they were given access to blood like in the natural environment. Overall, the findings clearly showed how *Aedes* mosquitoes are better adapted to urban environment.

The fecundity and longevity of the laboratory strain was the lowest in comparison to the other strains. This might be due to the environmental stress as the experiment was conducted under uncontrolled laboratory condition, where they are not used to. Abiotic factors such as temperature, relative humidity and rainfall affect the biology of the mosquito [34]. As reviewed by [34], temperature affects the development and survivorship of immature and adult stages of *Ae*. *albopictus*. It is also likely to influence biting rate because the durations of gonotrophic cycles are reduced at higher temperatures and at least a blood meal is accessed at each cycle [34], possibly affecting fecundity. Also, a study by [35] in Penang, Malaysia observed a strong correlation between rainfall and eggs of *Ae*. *albopictus* collected in urban and suburban areas. Hence the low performance of the laboratory strain compared to the field strains may be due to its less adaptability to the uncontrolled environmental conditions although it was carried out in the laboratory.

The blood meal of the mosquitoes in the various environments could differ depending on their host range. The rural environment is likely to have more diverse and broader host range compared to the suburban and urban areas where hematophagy is more likely to be on humans and in some instance’s pets. In neighboring Singapore, [36] reported that blood meals of *Ae*. *albopictus* were exclusively human in urban areas and though mostly human in rural areas, blood meals from animals (domestic and non-domestic) were also observed. However, in other geographic areas, anthropophagy is significantly more common in suburban than urban areas [37]. This shows that hematophagic activity can be on diverse and multiple hosts and varies in different urbanized and geographic settings, potentially impacting on fecundity of the mosquito. There is fewer vegetation as one progresses from rural to urban areas. Sugar (sucrose) is an important nutrient resource for mosquitoes and it has been shown to affect longevity of *Ae*. *albopictus* under laboratory conditions [38]. More sugar is likely to be consumed by mosquitoes in rural areas where vegetation is abundant compared to urban areas and this could affect hematophagic activity and consequent processes such as fecundity. Although body sizes of the strains were not assessed, it is likely they could affect biting rates, fecundity and longevity [38,39] of *Ae*. *albopictus* from the different urbanized areas if the variation in adult body size is significant.

## Conclusion

The results of this study indicate that urbanization might have a significant impact on the ecology and biology of *Aedes albopictus*. In the urbanizing and urbanized area, the changed environment probably serves as a suitable place for the growth and development of *Ae*. *albopictus;* the condensed human population produced more types of containers for larval habitats and more blood sources for adult reproduction. Warm climate may facilitate larval development; enhance vector survivorship and reproductive fitness. These might be the reasons for quick adaptation of *Ae*. *albopictus* in urban areas and thus susceptibility of this environment to the mosquito. Urbanization in Penang Island has exhibited strong positive effects on the reproductive and survivorship of adult mosquitoes through effects on the microclimatic condition of the mosquitoes, hence encourage dengue outbreak. The implications of these findings are that, if the current trends of urbanization continue in rural areas, *Ae*. *albopictus* could adapt to the changing environment and proliferate in rural areas, resulting in vast dengue outbreak in the future. These findings provide crucial information to the dengue vector control program and may be helpful to vector control experts in predicting outbreaks which could worsen if urbanization occurs without limits.

## Supporting information

S1 Fig (PNG)

S1 Table (PNG)
