# Supplementary figures and images for: Biting rhythm and demographic attributes of Aedes albopictus (Skuse) females from different urbanized settings in Penang Island, Malaysia under uncontrolled laboratory conditions
Source: PLoS One. 2020 Nov 11;15(11):e0241688. doi: 10.1371/journal.pone.0241688 (PMC7657491; doi:10.1371/journal.pone.0241688)

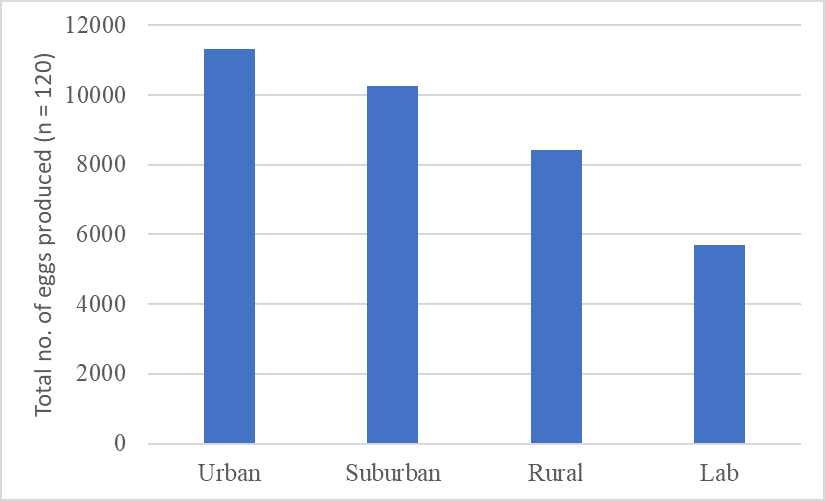

Supplement: S1 Fig — (PNG) [file pone.0241688.s001.png]

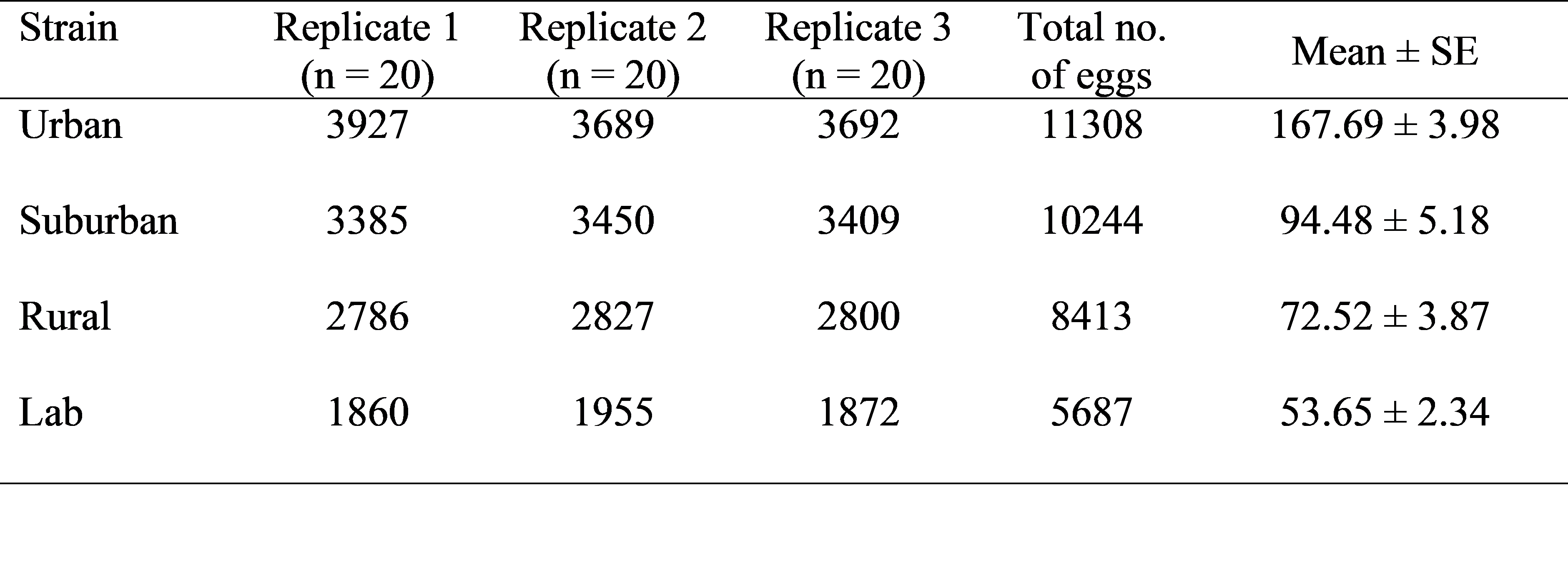

Supplement: S1 Table — (PNG) [file pone.0241688.s002.png]
